# Rapid Development of Breast Mass With Recurrent Episodes of Hypoglycemia Should Raise Suspicion of Breast Cancer With Insulinoma: A Case Report

**DOI:** 10.1002/cnr2.70243

**Published:** 2025-06-23

**Authors:** Tingting Liu, Lin Deng, Ruohan Su, Lin Ni, Zhiwei Wang, Wanqiu Xiong, Bing Wang, Sheng Huang

**Affiliations:** ^1^ Department of General Surgery Fuzong Clinical Medical College of Fujian Medical University Fuzhou China; ^2^ Department of General Surgery 900th Hospital of PLA Joint Logistic Support Force Fuzhou China; ^3^ Department of General Surgery Fuzong Teaching Hospital of Fujian University of Traditional Chinese Medicine Fuzhou China

**Keywords:** anhydrous alcohol ablation, breast tumors, case report delay in the condition and treatment, hypoglycemia, insulinoma

## Abstract

**Background:**

Breast cancer ranks first in the global incidence rate of malignant tumors in women. Despite the continuous emergence of new treatment methods, it remains a significant threat to the lives and quality of life of patients. Insulinoma, a rare pancreatic neuroendocrine tumor, causes pancreatic beta cells to over‐secrete insulin, disrupting the normal physiological feedback mechanism and leading to hyperinsulinemia. Studies have demonstrated that hyperinsulinemia can promote tumor development through various pathways, posing substantial challenges to tumor treatment. However, in previous studies, no successful treatment cases of breast cancer combined with insulinoma have been reported. Therefore, we present a case of metastatic breast cancer co‐occurring with insulinoma. The patient presented with a rapidly growing mass in the left breast accompanied by recurrent hypoglycemic symptoms. Following our treatment, the condition was significantly and effectively controlled.

**Case:**

A 57‐year‐old female with a rapidly enlarging mass in the left breast for over 1 year, currently measuring approximately 11 × 10 × 8 cm, with multiple enlarged and fused lymph nodes palpable in the ipsilateral axilla and bilateral neck. She had a history of recurrent hypoglycemic symptoms for 2 years, confirmed by laboratory tests, imaging examinations, fine needle aspiration biopsy (FNA), as metastatic breast cancer combined with insulinoma. On August 3, 2023, she underwent ultrasound‐guided alcohol ablation of the insulinoma and complete neoadjuvant therapy for breast cancer. The hypoglycemic symptoms disappeared and the tumor was rapidly and effectively controlled. Unfortunately, due to economic reasons, the patient refused further surgical treatment.

**Conclusion:**

The unique aspect of this case lies in the rapid growth of a breast mass over a one‐year period, accompanied by recurrent episodes of hypoglycemia. Following clinical evaluation, the diagnosis was metastatic breast cancer with concomitant insulinoma. To our knowledge, reports of similar cases are scarce. This case underscores the importance for clinicians to remain vigilant when encountering rapidly progressing cancers, particularly in identifying various factors that may contribute to cancer development. When multiple diseases coexist, it is crucial to recognize the interrelationships between these conditions and to adopt a multidisciplinary approach to treatment, thereby striving to provide the best personalized care for patients.

**Abbreviations**
AJCC: American joint committee on cancer
CEA: carcinoembryonic antigen
ESMO: European society for medical oncology
EUS‐EA: endoscopic ultrasound‐guided ethanol ablation
FNA: fine‐needle aspiration biopsy
GH: growth hormone
HER‐2: human epidermal growth factor receptor‐2
IGF: insulin‐like growth factor
IGF‐1: insulin‐like growth factor‐1
IGF‐1R: IGF‐1 receptor
IGF‐2: insulin‐like growth factor‐2
INSM1: insulinoma‐associated protein‐1
MDT: multidisciplinary team
NICTH: non‐islet cell tumor hypoglycemia
VEGF: vascular endothelial growth factor

## 1 Introduction

Breast cancer ranks first in the global incidence rate of malignant tumors in women and can be accelerated by various factors [1]. Insulinoma is a rare pancreatic neuroendocrine tumor that causes pancreatic beta cells to over‐secrete insulin, leading to hyperinsulinemia due to the loss of normal physiological feedback mechanisms [2]. Traditional studies [3, 4] have demonstrated that hyperinsulinemia disrupts the balance of the insulin‐growth hormone‐insulin‐like growth factor axis, shifting the insulin‐to‐growth hormone ratio toward insulin and away from growth hormone. This shift promotes energy storage and lipid synthesis while inhibiting lipid breakdown, potentially accelerating tumor growth. Additionally, hyperinsulinemia can activate signaling pathways by binding to insulin receptors and phosphorylating tyrosine residues, thereby promoting tumor cell proliferation and inhibiting apoptosis to facilitate tumor development [5].

Breast cancer combined with insulinoma is an extremely rare condition, and a prior literature review did not reveal similar cases. Hyperinsulinemia induced by insulinoma can accelerate the rapid development of breast cancer, presenting significant diagnostic and therapeutic challenges. Thoroughly investigating the medical history and conducting comprehensive examinations are crucial to avoid misdiagnosis and treatment delays. We report a case of metastatic breast cancer combined with insulinoma, characterized by rapid breast cancer progression and recurrent hypoglycemic symptoms. Here, we provide complete case information, diagnostic processes, and effective treatment methods for reference by clinical practitioners.

## 2 Case Report

### 2.1 Patient History

A 57‐year‐old female patient was admitted to the hospital due to the discovery of a lump in the left breast for over a year. In June 2022, a lump measuring approximately 2 × 1 × 1 cm was detected in the left breast. The lump was not associated with local tenderness, skin changes, or nipple discharge, and therefore, it did not receive immediate attention or treatment. Over the past year, the lump gradually increased in size, reaching approximately 11 × 10 × 8 cm, and was accompanied by mild pain, skin redness, and other symptoms. On July 25, 2023, the patient sought medical attention at our hospital's breast surgery department. Upon admission, a physical examination revealed smooth skin on both breasts without peau d'orange changes, and the skin temperature was normal. Multiple fused masses were palpable in the left breast, measuring approximately 11 × 10 × 8 cm. These masses were hard in texture, with unclear borders, poor mobility, and local skin redness without ulceration. No lumps were palpable in the right breast. Enlarged, fused lymph nodes were palpable in the left axilla, measuring approximately 2 × 2 × 1 cm, while none were palpable in the right axilla. Enlarged lymph nodes were palpable in the bilateral neck regions, with the largest measuring approximately 3 × 2 × 1 cm. The day after admission, blood glucose testing revealed a critical value of 1.91 mmol/L. Further inquiry into the medical history revealed that 2 years ago, the patient experienced two episodes of fainting due to hunger, accompanied by transient consciousness disturbances. Subsequently, the patient reported recurrent symptoms of dizziness, weakness, and cold sweats following periods of hunger, which could be relieved after eating. However, no treatment was sought.

### 2.2 Laboratory and Imaging Examinations

**FIGURE 1:**
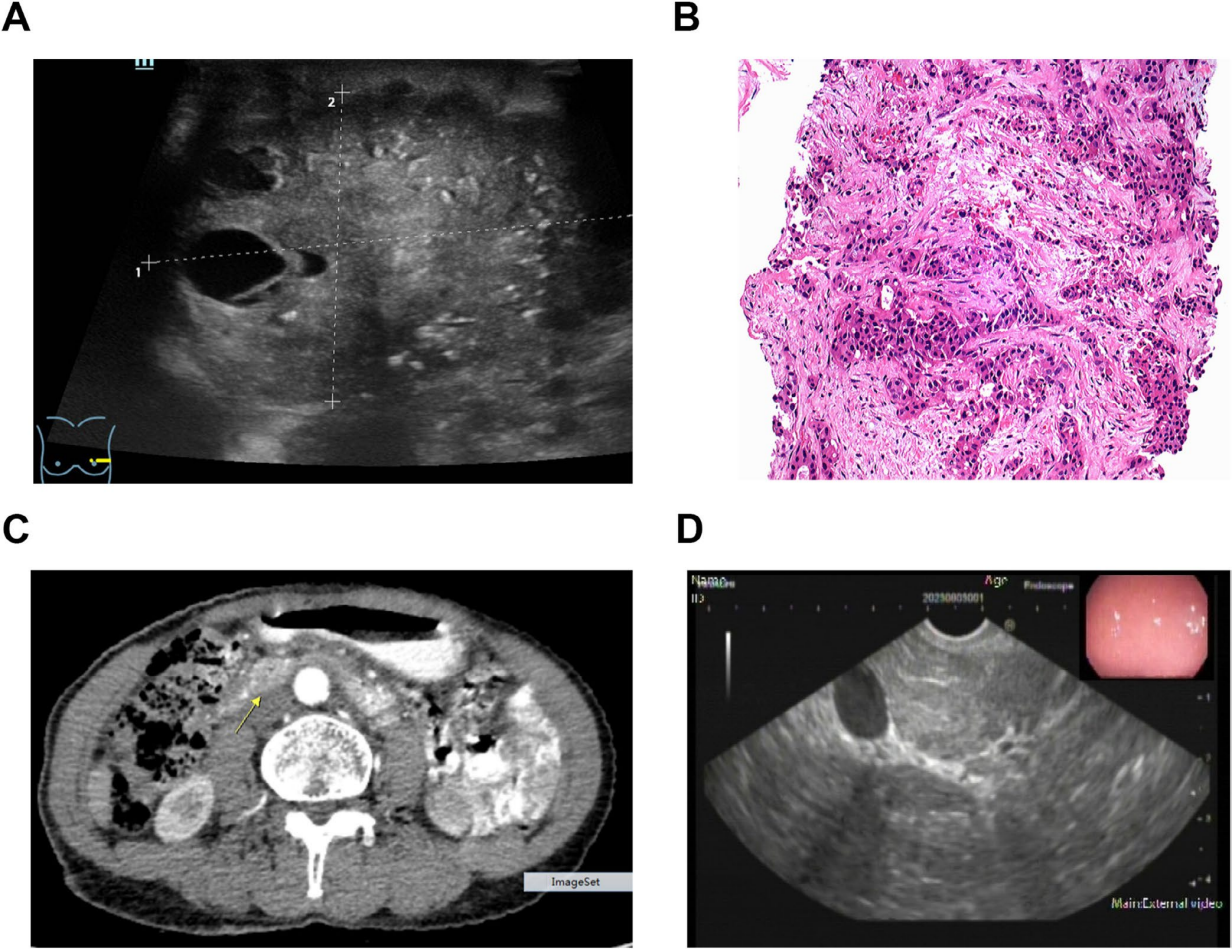
Images of relevant examinations at the time of admission of the patient. (A) US of left breast tumor. (B) HE staining of left axillary mass puncture pathology, HE×100. (C) CT insulinoma of mid‐abdomen, and (D) Insulinoma scintigraphy under endoscopic ultrasound.

On July 26, 2023, a breast color Doppler ultrasound (Figure 1A) and chest CT scan revealed a lobulated soft tissue shadow in the left breast with indistinct borders and multiple small calcifications within. Additionally, multiple fused enlarged lymph nodes were identified in the left axilla, measuring approximately 3.2 × 1.7 cm, suggestive of a malignant tumor with multiple lymph node metastases in the left axilla. On July 27, 2023, a breast MRI showed multiple nodular abnormal signals in the left breast, some of which were fused, with the largest measuring approximately 4.7 × 3.3 cm. These lesions had unclear margins and a lobulated appearance, indicative of breast cancer with multiple lymph node metastases in both axillae (BI‐RADS category V). Concurrently, an ultrasound‐guided fine needle aspiration biopsy was performed on the left breast mass, axillary mass, and right neck mass. Pathological results indicated infiltrating ductal carcinoma of the left breast with lymph node metastases in the left axilla and right neck. The tumor was positive for C‐erB‐2 (3+), Ki67 (20%), GATA‐3 (+++), and CK7 (++++), suggesting a non‐special type, histological grade II (tubular formation three points, nuclear grade two points, mitotic count two points, total score seven points), and a molecular subtype tending towards HER‐2 overexpression (Figure 1B). Abdominal ultrasound of the digestive and urinary systems and a whole‐body bone scan showed no significant abnormalities.

On July 28, 2023, a fasting blood glucose test result was 1.9 mmol/L, insulin level was 19.46 μU/mL, and C‐peptide level was 0.92 ng/mL. An abdominal CT scan conducted on the same day revealed a nodular enhancing lesion in the head of the pancreas, measuring approximately 2.0 × 1.2 cm, suggestive of a pancreatic islet cell tumor, with no other significant abnormalities observed. On August 1, 2023, an abdominal MRI showed a nodular lesion in the head of the pancreas, measuring approximately 1.5 × 1.2 cm, with moderate enhancement on contrast‐enhanced imaging, similar in enhancement degree to the pancreatic parenchyma, suggestive of a neuroendocrine tumor—pancreatic islet cell tumor (Figure 1C). Endoscopic ultrasound (EUS) indicated a slightly hyperechoic, round mass adjacent to the superior mesenteric vein in the head of the pancreas, with uniform internal echoes and abundant circular color flow, and hyperechoic borders locally, measuring approximately 17.1 × 10.8 mm in cross‐section, suggestive of the possibility of a pancreatic islet cell tumor (Figure 1D).

### 2.3 Diagnostic and Therapeutic Course

Based on the patient's clinical presentation and pathological examination, a diagnosis of metastatic breast cancer has been established. However, further investigation is required to determine the underlying cause of hypoglycemia, including the possibility of breast cancer pancreatic metastasis and non‐islet cell tumor hypoglycemia (NICTH) [6]. Therefore, we conducted a thorough examination of the patient, after numerous auxiliary tests and in combination with the Clinically specific manifestations and imaging results, we determined that the hypoglycemic symptoms were caused by an insulinoma. The final diagnosis was established as follows: (1) Left breast cancer (HER‐2 overexpression subtype) with left axillary lymph node and right cervical lymph node metastasis (staged as III C according to the eighth edition of the AJCC staging system [7], T3N3M0), (2) Insulinoma. Following the ESMO guidelines [8] for breast cancer management, neoadjuvant therapy for breast cancer was recommended. Due to the potential for high insulin levels caused by insulinoma to promote rapid progression of breast cancer, and the patient's recurrent hypoglycemic symptoms, a multidisciplinary team (MDT) discussion at our hospital suggested addressing the insulinoma with minimal invasiveness as a priority. This approach would allow the patient to receive neoadjuvant chemotherapy more effectively and promptly.

**FIGURE 2:**
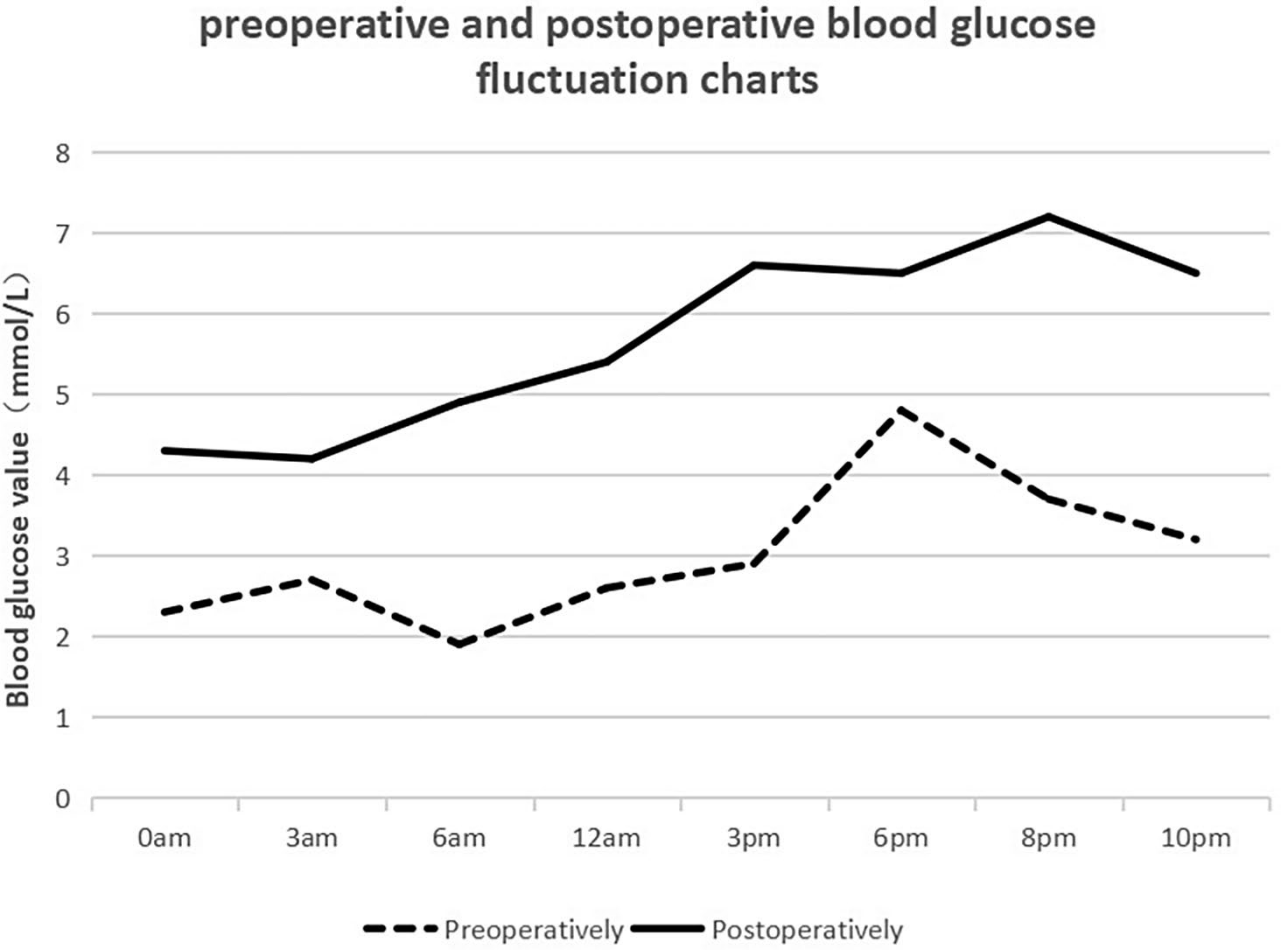
Comparison chart of preoperative and postoperative blood glucose fluctuations. The black dotted curve indicates the blood glucose fluctuations before the insulinoma surgery, and the black solid curve indicates the changes in blood glucose fluctuations after the insulinoma.

With the informed consent of the patient and her family, on August 3, 2023, under general anesthesia, endoscopic ultrasound‐guided ethanol ablation (EUS‐EA) was performed. Routine EUS scanning of the pancreatic lesion was conducted, avoiding blood vessels, and the puncture point was selected. A 22G puncture needle was inserted into the tumor, and after withdrawing the needle core, 2 mL of 98% anhydrous alcohol was injected into the tumor, resulting in a cloudy appearance and reduced blood flow signals on Doppler imaging. No active bleeding was observed at the puncture site after needle withdrawal. The following morning, the patient resumed oral intake, transitioning from a small, frequent semi‐liquid diet to a normal diet. Blood glucose monitoring ranged from 4.2 to 7.2 mmol/L, indicating good recovery compared to pre‐ablation levels (Figure 2). Plasma insulin and C‐peptide levels also significantly decreased (Table 1), and amylase levels gradually returned to normal. No complications such as bleeding, pancreatitis, or pancreatic fistula were noted. On the fourth day post‐operation, follow‐up EUS revealed a hyperechoic round mass adjacent to the superior mesenteric vein in the head of the pancreas, with uniform internal echoes, measuring 1.6 × 1.0 cm, indicating successful ablation.

**TABLE 1:** Changes in C‐peptide and insulin levels before and after surgery.

Time	Fasting C‐peptide	Fasting insulin	Postprandial C‐peptide	Postprandial insulin
	(ng/mL)	(μU/mL)	(ng/mL)	(μU/mL)
Preoperatively	0.92	19.46	5.20	> 300
Day 1 postoperatively	/	/	4.32	153
Day 3 postoperatively	/	/	3.15	134

Subsequently, on August 7, 2023, the patient underwent the first cycle of neoadjuvant therapy for breast cancer using the “paclitaxel + carboplatin + trastuzumab + pertuzumab” regimen, supplemented with symptomatic supportive treatments for gastric and liver protection, antiemesis, and immune enhancement. Within a week, a repeat blood routine and biochemical examination showed no significant abnormalities. The patient was discharged on August 9, 2023.

### 2.4 Follow‐Up

**FIGURE 3:**
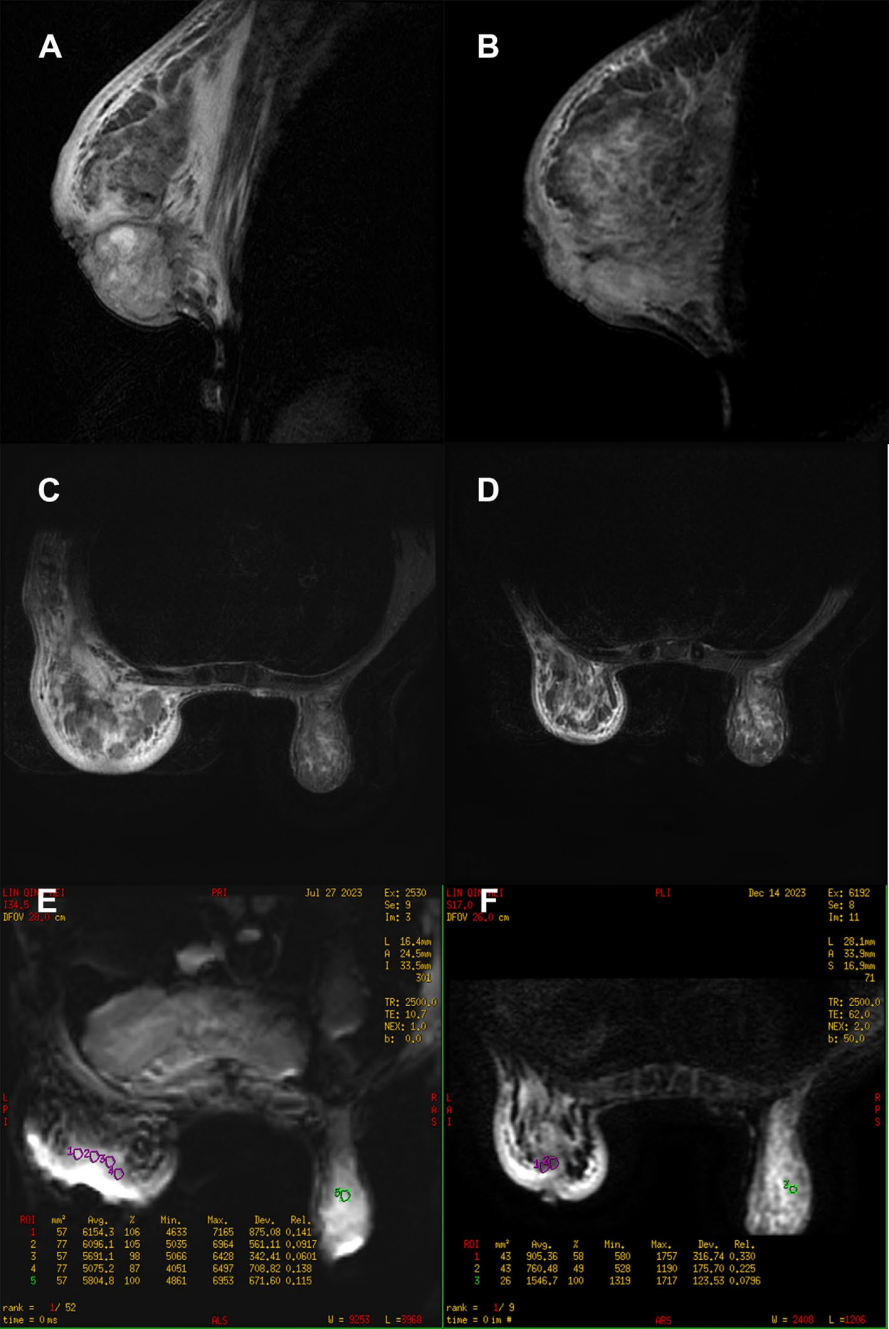
Comparison of breast MRI before and after six cycles of neoadjuvant therapy. The left (A, C, E) is the breast MRI image before neoadjuvant treatment, and the right (B, D, F) is the breast MRI image after neoadjuvant treatment.

From August 31, 2023, to December 16, 2023, the patient continued with five cycles of neoadjuvant therapy. On December 15, 2023, a follow‐up MRI showed a significant reduction in the lesion in the left breast, with the largest measuring approximately 1.1 × 1.0 cm, characterized by unclear margins and a lobulated appearance. The lymph nodes in the left axilla had also decreased in size, with the largest measuring approximately 1.0 × 0.9 cm, and no lesions were observed in the neck lymph nodes (Figure 3A–F). According to the eighth edition of the AJCC staging system, the stage was determined to be IIIA (T1N2M0). Tumor markers carcinoembryonic antigen (CEA) were 7.78 ng/mL and CA‐153 were 74.31 U/mL, showing a significant decrease compared to previous levels. Additionally, the patient's local breast pain, redness, and other symptoms had completely resolved; her mental and general condition had improved, and there were no further episodes of hypoglycemia, with blood glucose levels within the normal range. The overall treatment effect was significant. However, it is regrettable that the patient ultimately decided to forego further surgical treatment due to financial constraints. With the consent of the patient and their family (informed consent forms have been signed) and their active cooperation, we have decided to report this case to better guide clinical management.

## 3 Discussion

Breast cancer incidence is increasing year by year and is currently one of the major public health challenges globally [9]. Insulinoma is a rare pancreatic neuroendocrine tumor, with the majority being solitary and tending to be benign, but approximately 5%–10% exhibit malignant characteristics [10]. In our case, the patient presented with a rapidly growing breast mass and episodes of hunger‐related hypoglycemia characterized by abnormally elevated insulin levels. In our case, the patient presented with a rapidly growing breast mass and episodes of hunger‐related hypoglycemia characterized by abnormally elevated insulin levels. It was reported rare cases of NICTH previously [11], patients have presented with decreased levels of insulin and C‐peptide in the tumor, along with excessive secretion of insulin‐like growth factor‐2 (IGF‐2). IGF‐2 stimulates insulin receptors and increases glucose utilization, leading to hypoglycemia. Hypoglycemic symptoms resolved following the resection of the primary breast tumor. Hypoglycemia associated with paraneoplastic syndromes often manifests as irregular and rapid episodes, with no clear association with hunger state. Importantly, unlike insulinomas, these episodes typically do not coincide with elevated insulin levels but rather result from increased secretion of IGF‐2, leading to hypoglycemia [12]. Based on the patient's clinical presentation, we ruled out the possibility of hypoglycemia due to a paraneoplastic syndrome. Similarly, the case differed from those reported by Wang et al. [13], where breast cancer with synchronous pancreatic metastasis was managed with surgical resection of both primary and secondary tumors followed by chemotherapy, resulting in good disease control. Although both pancreatic metastasis from breast cancer and insulinomas can present radiologically as solitary, well‐circumscribed, smooth‐bordered, round or oval lesions, hypoglycemic symptoms caused by pancreatic metastasis from breast cancer are often less pronounced and may go unnoticed. These metastases are typically diagnosed through the identification of mammary‐specific markers, such as mammaglobin protein, expressed by the metastatic breast epithelial cells. In contrast, insulinomas are characterized by typical hypoglycemic symptoms. The significant fluctuations in blood glucose levels observed in this patient allowed us to largely rule out the possibility of pancreatic metastasis from breast cancer. Considered the patient's clinical features and extensive imaging evaluations, we arrived at a final diagnosis of metastatic breast cancer (TNM stage IIIC) with an insulinoma. After the insulinoma was ablated using anhydrous alcohol, the hypoglycemic symptoms immediately resolved, although no histopathological confirmation was obtained.

Patients with locally advanced breast cancer often cannot undergo direct surgical treatment and rely on neoadjuvant therapy to shrink the tumor before achieving a curative surgical goal. However, the effectiveness of neoadjuvant therapy varies greatly due to various reasons [14]. Insulinomas are characterized by the excessive secretion of insulin from the tumor, leading to hyperinsulinemia and subsequently causing hypoglycemia. Previous studies have demonstrated that hyperinsulinemia can increase lipid synthesis and inhibit lipolysis, thereby providing the nutrients and energy required by cancer cells, thus promoting rapid tumor growth. Insulin‐like growth factor‐1 (IGF‐1) and its receptor also play a crucial role in the development and progression of breast cancer. Research has shown that the binding of insulin and IGF‐1 to the insulin receptor can activate the PI3K/AKT/mTORC signaling pathway, stimulating cell proliferation and the transcription of metabolic genes, which promotes the growth of cancer cells and prevents apoptosis in breast cancer cells [15]. Some researchers have also proposed [16], hyperinsulinemia and IGF‐1 can alter the cell cycle regulation via the RAS/MAPK/ERK pathway, thereby promoting the growth, differentiation, and proliferation of tumor cells. Additionally, IGF‐1 and insulin can upregulate the expression of vascular endothelial growth factor (VEGF), increasing tumor angiogenesis and further promoting tumor growth [17]. Furthermore, the IGF‐1 receptor (IGF‐1R) can activate the Ras/Raf/MEK/ERK signaling pathway, enhancing the expression of cyclin D1 and accelerating the G1/S phase transition, thus promoting the proliferative capacity of breast cancer cells [18]. Recent studies have also identified neuroendocrine differentiation in non‐special type invasive breast cancer as a rare finding. Neuroendocrine differentiation markers, such as insulinoma‐associated protein‐1 (INSM1), encoded by the insulinoma‐associated protein‐1 gene, can promote breast cancer development by regulating C‐MYC [19]. In summary, insulinomas and IGF‐1 play a significant role in the development and progression of breast cancer, potentially affecting the efficacy of neoadjuvant therapy for breast cancer. Therefore, after a MDT discussion, it was decided to initially treat the insulinoma with the least invasive approach.

The treatment of neuroendocrine tumors mostly relies on surgical intervention, including open surgery and laparoscopic surgery. However, both methods have characteristics such as high trauma, long recovery time, and multiple complications. Anne et al. [20] studied 80 patients with benign insulinoma who underwent surgical treatment and found a postoperative pancreatic fistula rate of 44%. EUS‐EA is an interventional treatment method that involves the local injection of drugs into the lesion through a fine needle to induce tissue necrosis. Anhydrous alcohol acts on local tissues by causing protein denaturation, vascular destruction, local thrombosis, tissue necrosis, and the destruction of tumor cells. This process leads to inflammation and fibrotic reactions, thereby maximizing the elimination of tumor cells. A recent large multicenter study demonstrated that [21], compared to surgical treatment, EUS‐guided intervention therapy appears to be safer and more effective, with significantly fewer postoperative complications. This finding suggests that EUS‐guided intervention therapy may become the first‐line treatment for sporadic insulinoma.

After careful consideration, it was ultimately decided to perform ultrasound‐guided alcohol ablation for the treatment of the insulinoma. The patient recovered well postoperatively without any adverse complications and quickly proceeded to receive neoadjuvant therapy for breast cancer. Following six cycles of treatment, the patient met the criteria for complete resection surgery. However, throughout the course of diagnosis and treatment, we undoubtedly faced numerous challenging decisions. Initially, based on the principles of disease management, the treatment of breast cancer should have been prioritized. At that stage, without a comprehensive understanding of the interrelationships between the diseases, we overlooked the potential promoting effect of the insulinoma on the progression of breast cancer. This oversight might have posed long‐term challenges for the subsequent treatment of breast cancer. Furthermore, determining the optimal approach for treating the insulinoma presented another significant challenge. Current research has shown that EUS‐EA has achieved notable success due to its safety, effectiveness, and minimal invasiveness, making it our preferred option. However, this technique is still under active investigation in many developed countries and regions. It requires extremely high technical proficiency from the operator, unlike more established traditional surgical methods, and thus must be approached with caution. After MDT discussions, we received valuable input from various specialties. This collaboration not only provided a more comprehensive understanding of the coexistence of the two diseases but also connected us with highly skilled practitioners experienced in EUS‐EA. Ultimately, through the collective efforts of all disciplines, we formulated the best individualized treatment plan for the patient. Although the patient ultimately opted against surgical treatment due to financial constraints, the personalized treatment regimen clearly yielded significant therapeutic benefits.

## 4 Conclusion

Through an in‐depth study of this case, we believe that it is essential for clinicians to have a comprehensive understanding of the patient's condition and the interrelationships between different diseases, particularly when endocrine tumors coexist with cancer. Given that endocrine tumors often cause metabolic disorders, and that endocrine metabolism is closely linked to the development and progression of cancer, these factors must be given due attention. Moreover, the importance of multidisciplinary collaboration in diagnosis and treatment becomes evident. Through communication among multiple disciplines, a more thorough understanding of the patient's condition can be achieved, leading to the formulation of personalized treatment plans that maximize therapeutic benefits. To date, we have not encountered similar cases. We hope that future research will be more extensive, fully exploring the interrelationships between metabolism, cancer, and other diseases. This will further validate our treatment concepts and strategies, thereby better guiding clinical practice.

## Author Contributions

T.L. and L.D. designed the study plan and collected clinical data. L.N. and R.S. analyzed the data and made graphs and tables. Z.W. and W.X. tracked and refined the manuscript revisions. T.L., B.W., and S.H. drafted the manuscript and made significant revisions to it. All authors contributed to the review and further revision of the manuscript and approved the final version for submission.

## Ethics Statement

This case report was written and published after obtaining informed consent from the patient and their family, and with their cooperation. The patient has signed the relevant informed consent form (20231216), and there are no ethical issues involved.

## Consent

The authors have nothing to report.

## Conflicts of Interest

The authors declare no conflicts of interest.

## Data Availability

The data that support the findings of this study are available from the corresponding author upon reasonable request.
